# Does Inflammation Mediate the Obesity and BPH Relationship? An Epidemiologic Analysis of Body Composition and Inflammatory Markers in Blood, Urine, and Prostate Tissue, and the Relationship with Prostate Enlargement and Lower Urinary Tract Symptoms

**DOI:** 10.1371/journal.pone.0156918

**Published:** 2016-06-23

**Authors:** Jay H. Fowke, Tatsuki Koyama, Oluwole Fadare, Peter E. Clark

**Affiliations:** 1 Departments of Medicine and Urologic Surgery, Vanderbilt University Medical Center, Nashville, TN, United States of America; 2 Department of Biostatistics, Vanderbilt University Medical Center, Nashville, TN, United States of America; 3 Department of Pathology, University of California San Diego, San Diego, CA, United States of America; 4 Department of Urologic Surgery, Vanderbilt University Medical Center, Nashville, TN, United States of America; Oklahoma University Health Sciences Center, UNITED STATES

## Abstract

**Background:**

BPH is a common disease associated with age and obesity. However, the biological pathways between obesity and BPH are unknown. Our objective was to investigate biomarkers of systemic and prostate tissue inflammation as potential mediators of the obesity and BPH association.

**Methods:**

Participants included 191 men without prostate cancer at prostate biopsy. Trained staff measured weight, height, waist and hip circumferences, and body composition by bioelectric impedance analysis. Systemic inflammation was estimated by serum IL-6, IL-1β, IL-8, and TNF-α; and by urinary prostaglandin E2 metabolite (PGE-M), F2-isoprostane (F2iP), and F2-isoprostane metabolite (F2iP-M) levels. Prostate tissue was scored for grade, aggressiveness, extent, and location of inflammatory regions, and also stained for CD3 and CD20 positive lymphocytes. Analyses investigated the association between multiple body composition scales, systemic inflammation, and prostate tissue inflammation against BPH outcomes, including prostate size at ultrasound and LUTS severity by the AUA-symptom index (AUA-SI).

**Results:**

Prostate size was significantly associated with all obesity measures. For example, prostate volume was 5.5 to 9.0 mls larger comparing men in the 25^th^ vs. 75^th^ percentile of % body fat, fat mass (kg) or lean mass (kg). However, prostate size was not associated with proinflammatory cytokines, PGE-M, F2iP, F2iP-M, prostate tissue inflammation scores or immune cell infiltration. In contrast, the severity of prostate tissue inflammation was significantly associated with LUTS, such that there was a 7 point difference in AUA-SI between men with mild vs. severe inflammation (p = 0.004). Additionally, men with a greater waist-hip ratio (WHR) were significantly more likely to have severe prostate tissue inflammation (p = 0.02), and a high WHR was significantly associated with moderate/severe LUTS (OR = 2.56, p = 0.03) among those participants with prostate tissue inflammation.

**Conclusion:**

The WHR, an estimate of centralized obesity, was associated with the severity of inflammatory regions in prostate tissue and with LUTS severity among men with inflammation. Our results suggest centralized obesity advances prostate tissue inflammation to increase LUTS severity. Clinically targeting centralized fat deposition may reduce LUTS severity. Mechanistically, the lack of a clear relationship between systemic inflammatory or oxidative stress markers in blood or urine with prostate size or LUTS suggests pathways other than systemic inflammatory signaling may link body adiposity to BPH outcomes.

## Introduction

The diagnosis of benign prostatic hyperplasia (BPH) is often in response to the development of lower urinary tract symptoms (LUTS), including urinary hesitancy, urgency, and frequency. These symptoms are among the most common morbidities associated with aging in men [1–4]. Medical treatment options include α-adrenergic antagonists or 5-α reductase inhibitors, however about one-third of men with LUTS do not respond to either treatment approach [5]. Patients who are resistant to treatment, or who become resistant to treatment over time, will become candidates for surgical intervention to reduce LUTS severity. Further understanding the causes of LUTS will guide interventions to prevent LUTS or increase sensitivity to medical treatment.

Regions of chronic inflammation are common across the stroma and glandular epithelium of human prostate tissue [6], with the potential to drive cell proliferation and angiogenesis [7]. Analysis of data and biospecimens from the Medical Therapies of Prostatic Symptoms (MTOPS) study found inflammatory infiltrates associated with a larger prostate volume and LUTS progression [7–9]. Similarly, chronic inflammation was associated with LUTS severity in the Reduction by Dutasteride of Prostate Cancer Events (REDUCE) trial [10]. Obesity is also one of the more consistent risk factors for BPH [11, 12]. For example, analysis of data from the Prostate Cancer Prevention Trial (PCPT) found increased body mass index (BMI) significantly associated with more severe LUTS, while a greater waist-hip ratio (WHR) was marginally associated with moderate to severe LUTS (American Urologic Association Symptom Index (AUA-SI) ≥ 15: RR_(BMI≥30)_ = 1.30, 95% CI (1.08, 1.47), RR_(WHR≥1.05)_ = 1.30, 95% CI (0.95, 1.78)) [13]. Similarly, a larger waist circumference (WC) was significantly associated with BPH surgery in the Health Professionals Follow-up cohort (RR_(WC>39–43 cm)_ = 1.46 (1.07, 2.01); RR_(WC>43 cm)_ = 1.64 (1.07, 2.54), p-trend = 0.003)[14]. Prostate enlargement is a secondary component cause of LUTS reflecting increased prostate cell proliferation and benign hyperplasia in prostate tissue. Our prior research, as well as analysis of the Olmstead County Study and the Baltimore Longitudinal Study of Aging, found obese men had a significantly larger prostate size compared to non-obese men [15–17]. Obesity is well-known to be linked with cardiovascular disease and other inflammation-related diseases [18, 19], and these prior BPH studies suggest obesity in some way generates an environment conducive to prostate enlargement and LUTS progression.

We hypothesize that obesity drives a state of chronic systemic inflammation, leading to prostate tissue immune cell infiltration, tissue remodeling, hyperplasia, benign prostatic enlargement, increased LUTS severity, and clinical BPH. Regions of hypoxia and cell necrosis may form within adipose tissue as the amount of adipose tissue increases. Macrophages and other immune cells infiltrate the adipose tissue mass in response to necrosis, resulting in increased cytokine levels and generation of reactive oxygen species (ROS) [18, 19], and a state of chronic systemic inflammation that may support immune cell infiltration into the prostate. Additional proinflammatory cytokines may be released into the prostate stroma, triggering stromal cell proliferation and culminating in prostate enlargement or increased LUTS severity [9, 20, 21].

**Fig 1:**
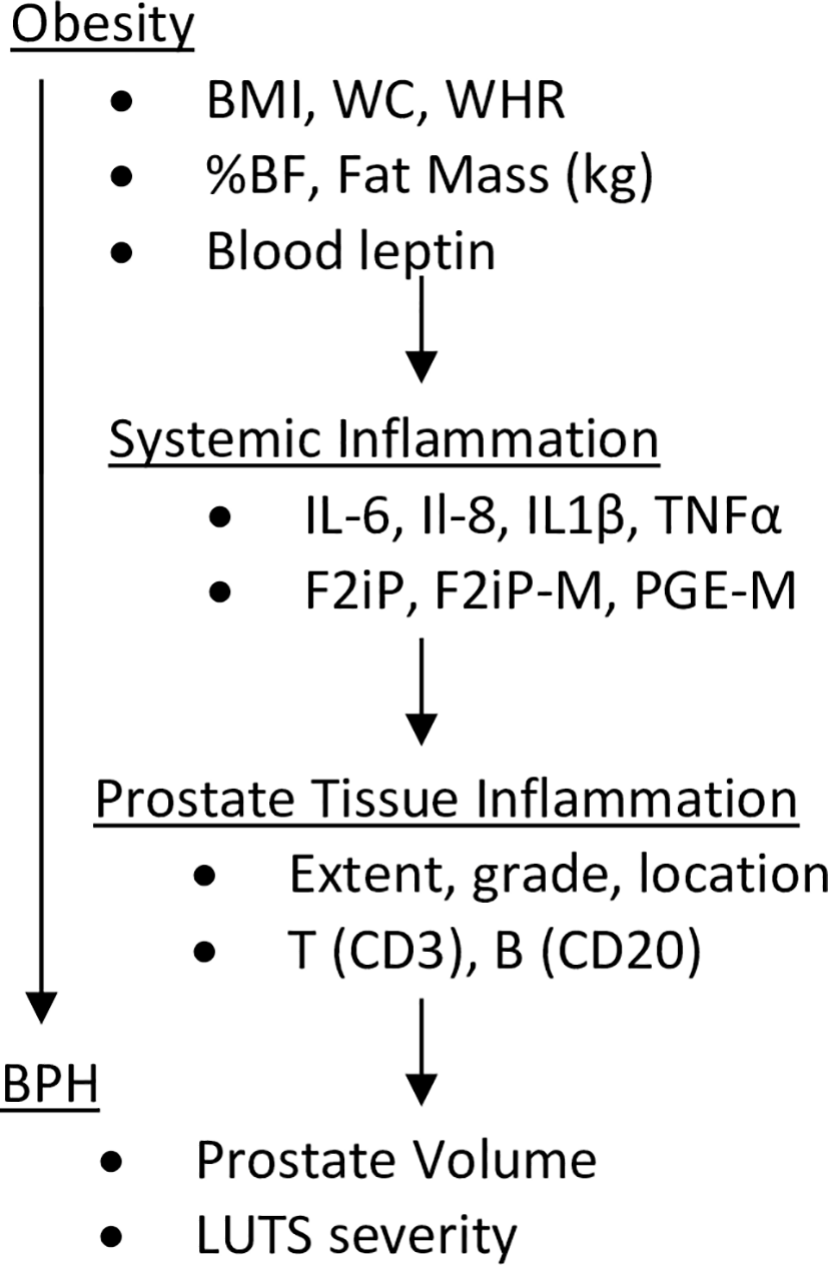
Obesity and BPH: Conceptual Approach. Excess body adiposity or a centralized body fat deposition may lead to increased levels of circulating proinflammatory cytokines and systemic oxidative stress. Proinflammatory signaling may facilitate increased immune cell invasion and prostate tissue inflammation, leading to prostate enlargement and greater LUTS severity.

Fig 1 summarizes the conceptual approach of this study, with prostate size and LUTS severity serving as BPH outcomes. Analyses investigate the relationship between these outcomes with obesity and body composition measures; blood and urinary markers of inflammation and oxidative stress; the extent, grade, aggressiveness, and location of prostate tissue inflammation; and T and B cell infiltration in prostate tissue. This comprehensive approach in a single study is unique, and allows us to investigate the links between obesity, systemic inflammation, prostate tissue inflammation, and BPH outcomes.

## Materials and Methods

### Study Participants

Participants were enrolled as a part of the Nashville Men’s Health Study, a multi-centered, rapid-recruitment protocol initiated in 2002 and targeting men seeking a diagnostic prostate biopsy at Vanderbilt University Medical Center, the Tennessee Valley Veterans Administration Medical Center, and Urology Associates—a large community urology practice [22]. Exclusion criteria included age less than 40 years, a prior prostate cancer diagnosis, prior prostate surgery, use of androgen supplementation, or English language insufficient for informed consent. Approximately 90% of eligible men approached for recruitment consented to participate, and a single pathologist reviewed clinical biopsies for consistency. All protocols were approved by IRBs at Vanderbilt University and the Tennessee Valley Veterans Administration, and all subjects provided written informed consent prior to data collection.

### Medical Chart Review and AUA-SI

Data abstraction from urology, surgery, and pathology medical reports included PSA test history, the number of prior biopsies, number of prostate cores collected at biopsy leading to recruitment, prostate volume (ml) at biopsy ultrasound, and use of **α-**blockers or 5-**α**reductase inhibitors. A structured research questionnaire was also administered at the time of recruitment that included the American Urological Association Symptom Index (AUA-SI) [23].

### Obesity and Body Composition

Weight (kg) (no shoes, hospital gown) was measured on a calibrated scale, and height (within 0.1 cm) was measured by stadiometer at the time of recruitment by trained staff. Waist and hip circumferences are measured using an anthropometric tape measure with built-in tension meter (Gullick II) to ensure a consistent tape tension during measurement. Waist circumference was measured at the narrowest part of the torso, and hip circumference was measured at the maximum posterior extension of the buttocks. Staff performed two measurements at each site in rotational order, with a third measurement if the first two differ by more than 1 cm. The collected circumference measures were averaged prior to analysis. Body composition, including total fat mass (kg), total lean mass (kg), and % body fat (%BF), was measured by bioelectric impedance analysis (BIA) (Tanita Corporation, Arlington Heights, IL). Participants were without socks, wearing a clinical robe, and provided drinking water to ensure hydration during BIA measurement.

### BPH Biomarker Sub-study

To focus on the non-malignant causes of prostate enlargement and LUTS, we restricted this analysis to NMHS participants with a negative prostate biopsy. We excluded patients diagnosed with prostate cancer, high-grade prostatic intraepithelial neoplasia (HGPIN), atypical small acinar proliferation lesion, or other results suspicious for prostate cancer. We further restricted to participants recruited between 2009 and 2012 and who completed the AUA-SI for LUTS severity. Participants with an AUA-SI greater than 30 were also excluded to prevent these few participants from having strong influence on the analysis. The final study population included 191 men without PC at biopsy.

### Prostate Tissue Inflammation

Prior to scoring prostate tissue cores for inflammation, we conducted a pilot study to support feasibility by comparing inflammation in biopsy cores collected at the apex, mid, and base prostate from 5 patients. We found inflammation levels in the mid-prostate were comparable to that of the apex, and only slightly higher than the base, and therefore focused analyses on the mid-region as representative in order to support the feasibility of analysis in our study sample. A broader analysis comparing inflammation levels in cores vs. blocks found similar results [24]. Therefore, our final prostate tissue inflammation scoring was performed on the mid-prostate on both the left and right sides from each patient.

Our goal was to identify and score regions of moderate to severe chronic inflammation. The approach was adapted from the consensus criteria developed by Nickel, the North American Chronic Prostatitis Collaborative Research Network, and the International Prostatitis Collaborative Network [25]. A similar strategy was recently used by Song and colleagues [26] and additionally adapted by Irani and colleagues [27, 28]. Tissue samples were stained by hematoxylin and eosin (H&E), and images were taken to capture the entire length of each stained prostate core using the Clearview Software package from Olympus. Images at 20X were sufficient to characterize inflammatory regions in tissue for most slides, while an increase magnification to 40X was occasionally necessary. The glass slides, as well as resultant images, were reviewed in a blinded manner by an experienced pathologist (author: OF) without knowledge of the patients’ prostate size or LUTS severity. Within each image, regions of inflammation (ROI) were identified for further analysis and processing. These ROI included confluent sheets of inflammatory cells, with or without tissue destruction or lymphoid nodule or follicle formation. The ROI approach enabled us to create cumulative inflammation scores across all images for all cores from the left and right for each patient, as well as provide a means to select a segment of tissue with levels of inflammatory cells sufficient to analyze and score.

The inflammation in each ROI was further scored on four parameters:

1. Location: Anatomic location (glandular, periglandular, stromal), with “glandular” infiltrates being within the epithelia or lumina of ducts/glands, “periglandular” infiltrates being in stroma, centered around ducts/glands for a distance of no more than 50 μm, and “stromal” infiltrates being within stroma, not centered around ducts and glands and being 50 μm or more from them [25]. With few ROIs categorized as strictly stromal, we combined periglandular + stromal ROIs for comparison to glandular ROIs.
2. Aggressiveness: For those ROIs identified as “glandular”, the level of inflammatory cell infiltration and disruption of the glandular epithelia was further scored on a 4-tiered scale using the criteria of Irani et al. (0: no contact between inflammatory cells and glandular epithelium; 1: minimal contact between inflammatory cells and glandular epithelium, without overt epithelial disruption; 2: interstitial inflammatory infiltrate associated with a clear but limited (less than 25% of examined material) glandular epithelium disruption; 3: glandular epithelium disruption on more than 25% of the examined material [28].
3. Grade: The ROI grade represents an estimation of the inflammatory cell density in each ROI. We applied the 3-tiered system proposed by Nickel and colleagues [25], and assigned a grade to each ROI as follows: 1/mild: scattered individual inflammatory cells, predominantly separated by intervening spaces (estimated density of less than 100 cells/mm^2^); 2/moderate: sheets of inflammatory cells, predominantly confluent, without lymphoid follicle formation or overt tissue destruction (estimated density of 100–500 cells/mm^2^); 3/severe: confluent sheets of inflammatory cells with tissue destruction and/or lymphoid follicle formation, (estimated density of greater than 500 cells/mm^2^).
4. Extent: The extent of inflammation in tissue from each patient was calculated as the total area of all ROI across all images for that patient divided by the total tissue area scored for that patient x 100, to yield as the percent of all analyzed cores for a given patient with inflammation.

### Immune Cell Infiltration

We measured CD3, a marker of T cell infiltration, and CD20, a marker of B cell infiltration, on an automated immunostainer in the Vanderbilt Translational Pathology Core. Duplicate slides from the mid-prostate on the left and right were stained for each patient. Images of stained slides were generated in the Vanderbilt Digital Histology Shared Core using an Aperio Scanscope XT®. Percentage of tissue with immune cell infiltration was calculated as the number of positive pixels for CD3 or CD20 divided by the total number of pixels in the scorable tissue space. Representation of areas with corpus amylacea, crushed or stained margins, or damaged tissue removed from the scorable tissue space either programmatically or manually.

This measurement is very precise as a quantitative measure of immune cell staining, however it cannot differentiate intense focal staining in a portion of the core vs. diffuse moderate staining involving the whole core. Since it is not clear that these two situations are indeed of equal pathological importance, and because obesity may advance a pre-existing inflammatory state rather than initiate an original inflammation locus, a second series of measurements examined the intensity of positive staining. Images of cores were divided into 500 x 500 micron (0.5 x 0.5 mm) square regions, and within each region we measured the percent composition of the positive stain. The level of staining was then sorted in ascending order of percent positive staining within each region across the entire population of biopsies for each patient, providing an index of each patient’s maximal CD3 or CD20 positive staining.

### Urine and Blood Biomarkers of Inflammation and Oxidative Stress

Serum cytokine levels were measured in the Vanderbilt Hormone Assay Core Lab using a magnetic bead-based multi-analyte panel on the Luminex bead-based assay (Millipore Inc., Billerica, MA). Serum leptin, an adipocytokine and a biomarker of body adiposity, was also assayed on the Luminex platform. All assay coefficients of variation were less than 10%. Urinary prostaglandin E2- metabolite (PGE-M) is an index of COX activity and endogenous PGE_2_ levels in humans, and was measured by liquid chromatography/tandem mass spectrometric (LC/MS) method [29]. The lower limit of detection of PGE-M was 40 pg, a level approximately 100-fold below what is found in normal human urine. The coefficient of variation for samples analyzed in multiple batches was 7.2% and the assay accuracy was 93% (*n* = 4 separate experiments [29]). F2-isoprostane measurement provides a measure of *in vivo* lipid peroxidation as an estimate of systemic oxidative stress [30, 31]. To avoid concern that unmetabolized isoprostanes may be artificially generated *in vitro* in biological fluids by autoxidation or that the level may be significantly affected by the renal isoprostane production, we also measured the F2-isoprostane metabolite (2,3-dinor-5,6-dihydro-15-F_2t_-IsoP or F2iP-M)[30–32]. F2iP and F2iP-M were measured by gas chromatography/negative ion chemical ionization mass spectrometry (GC/NICI MS) [30, 31]. Urinary markers were standardized to urinary creatinine prior to analysis to control for differences in urine volume.

### Statistical Analysis

Patient characteristics were summarized with median and quartiles for continuous variables and frequency and percent for categorical variables. Multivariable linear regression was used to estimate the association between each assessment of obesity (i.e., BMI, WC, WHR, leptin), tissue inflammation (i.e., grade, extent, aggressiveness, location), and immune cell infiltration (i.e., CD3 and CD20; average and maximum) with LUTS severity (i.e., score on AUA-SI) or prostate volume (mls) at ultrasound. The number (n) may vary somewhat across biomarker measures due to assay failure or lack of sample. In each model, age and prior BPH medication were included as additional covariates. Serum and urinary markers were log-transformed prior to analysis, and then transformed back to the original scale for presentation. Interaction between the variable of interest and BPH medication was considered, and results are included in S1 File. The interaction term was not statistically significant in the vast majority of analyses, while those instances were results differed by BPH treatment status are indicated in the text. Beta coefficients from regression models are reported to provide an overall effect estimate. We also determine difference in AUA-SI or prostate volume that corresponds to change in the obesity measure from the 25^th^ percentile to the 75^th^ percentile, providing a more easily interpreted measure of effect that is within the range of the data. Multivariable logistic regression was also used in the analyses investigating the association WHR and LUTS associations. Statistical significance was defined as p ≤ 0.05, and interpreted in the context of our conceptual model and hypotheses (Fig 1). All analyses were conducted with R (http://www.R-project.org/).

## Results

### Study Population

The median age of study participants was 65 years (Table 1). Most were married, self-defined as white, and had a PSA level of 4 ng/ml or more. About 34% had a BMI of 30 or more, and median %BF was 27.4%. Indices of centralized fat deposition include WC and WHR, with median values of 41 cm and 1.03, respectively. About 29% of men were taking medications to treat BPH, and the median prostate volume and AUA-SI score were 48 mls and 9, respectively.

**Table 1:** Study Population Characteristics.

**Factor (continuous)**	**N**	**Median**	**Quartiles**
Age (years)	191	65.0	60.5, 70.0
Height (cm)	191	175	172, 180
BMI	191	28.5	25.9, 30.8
WC (cm)	191	41.0	37.5, 43.3
WHR	191	1.027	0.976, 1.071
Fat mass (kg)	182	24.5	18.8, 30.4
Lean mass (kg)	182	62.3	56.9, 66.7
% body fat	182	27.4	24.0, 31.9
PSA (ng/ml)	189	4.5	3.2, 6.1
AUA-SI	191	9	4, 15
Prostate volume (mls)	186	48.0	34.7, 66.8
**Factor (categorical)**	**Level**	**N**	**%**
Race	African American	6	3%
	White	185	97%
Marital Status	Married	170	89%
	Widowed	6	3%
	Divorces/Single	15	8%
BPH treatment	Yes	55	29%
LUTS (AUASI score)	0–7	88	46%
	8–20	79	41%
	21–30	24	13%
Prostate Volume	Less than 40 mls	64	34%
	40–59 mls	64	34%
	60 mls or more	58	32%

### Body Composition and Prostate Volume or LUTS severity

We first considered the potential interaction between obesity and use of medications for BPH, but found almost no interactions to be statistically significant in predicting either prostate volume or LUTS severity (Tables A and B in S1 File). Thus, analyses investigating obesity and BPH derived from models that controlled for age and BPH treatment, unless otherwise specified. Increasing prostate volume was significantly associated with BMI, WHR, WC, %BF, Total Fat Mass, and Total Lean Mass (Table 2). For example, prostate volume was 7.19 mls larger among men with a BMI of 30.8 compared to men with a BMI of 25.9. Similarly, prostate volume was between 6.59 to 9.04 mls higher, on average, comparing men in the lowest vs. the highest quartile of body fat mass or lean mass composition scores, respectively. Serum leptin, an adipokine, was also significantly associated with prostate enlargement (p = 0.008, Table 3). In contrast, obesity measures including body composition and leptin levels were not significantly associated with LUTS severity.

**Table 2:** Association between Body Composition and Prostate Volume or Lower Urinary Tract Symptom Severity (LUTS).

**Prostate volume**									
**Obesity**	**Unit**	N*	Beta***	**P**	**Obesity Quartiles**		**PV difference**^**#**^	**95% CI**	
BMI	kg/m^2^	186	1.47	0.0003	25.9	30.8	7.19	3.30,	11.07
WHR*	ratio	186	0.55	0.033	97.6	107.1	5.22	0.41,	10.02
WC	cm	186	1.27	0.0006	37.5	43.3	7.31	3.15,	11.46
Height	cm	186	0.53	0.068	172	180	4.65	-0.34,	9.65
% Body Fat	%	177	0.70	0.021	24.0	31.9	5.48	0.85,	10.10
Lean Mass	kg	177	0.92	0.0001	56.9	66.7	9.04	4.57,	13.50
Fat Mass	kg	177	0.57	0.0025	18.8	30.4	6.59	2.34,	10.83
**LUTS**									
**Obesity**	**Unit**	N*	**Beta**	**P**	**Obesity Quartiles**		**LUTS difference**^**#**^	**95% CI**	
BMI	kg/m^2^	191	0.096	0.39	25.9	30.8	0.47	-0.61,	1.55
WHR*	ratio	191	0.013	0.86	97.6	107.1	0.12	-1.20,	1.44
WC	cm	191	0.055	0.59	37.5	43.3	0.32	-0.84,	1.47
Height	cm	191	0.026	0.74	172	180	0.23	-1.13,	1.58
% Body Fat	%	182	0.059	0.47	24.0	31.9	0.46	-0.79,	1.71
Lean Mass	kg	182	0.061	0.36	56.9	66.7	0.60	-0.68,	1.88
Fat Mass	kg	182	0.051	0.33	18.8	30.4	0.58	-0.59,	1.75

* N may vary due to missing values.

** WHR * 100

*** Beta–coefficient from linear regression model adjusted for age and treatment for BPH

# Difference in PV or LUTS between the quartile values of each body composition measurement.

**Table 3:** Inflammatory Biomarkers and Prostate Volume and Lower Urinary Tract Symptom Severity.

**Prostate Volume**										
	Marker	Unit	N*	Beta**	P	Marker Quartiles^#^		PV difference	95% CI	
Serum	IL-6	pg/ml	158	0.82	0.70	1.90	6.17	0.96	-4.00,	5.93
	IL-8	pg/ml	158	-3.84	0.29	2.91	5.35	-2.34	-6.68,	2.00
	TNF-α	pg/ml	158	3.36	0.41	2.83	5.03	1.93	-2.66,	6.52
	IL-1β	pg/ml	158	-2.12	0.42	0.92	2.59	-2.19	-7.52,	3.14
	Leptin	pg/ml	158	5.58	0.008	4144	13417	6.56	1.70,	11.42
Urine	PGE-M	ng/ml	160	-1.92	0.30	8.67	38.60	-2.86	-8.34,	2.62
	F2iP	ng/ml	156	-2.63	0.14	0.92	4.98	-4.43	-10.39,	1.52
	F2iP-M	ng/ml	158	-2.02	0.23	0.39	2.05	-3.36	-8.87,	2.14
**LUTS**										
	Marker	Unit	N	Beta	P	Marker Quartiles^#^		LUTS difference	95% CI	
Serum	IL-6	pg/ml	162	-0.99	0.078	1.90	6.17	-1.17	-2.47,	0.13
	IL-8	pg/ml	162	-1.79	0.061	2.91	5.35	-1.09	-2.24,	0.05
	TNF-α	pg/ml	162	0.35	0.73	2.83	5.03	0.20	-0.95,	1.35
	IL-1β	pg/ml	162	-1.39	0.043	0.92	2.59	-1.44	-2.83,	-0.04
	Leptin	pg/ml	162	0.38	0.50	4144	13417	0.44	-0.85,	1.74
Urine	PGE-M	ng/ml	164	-0.43	0.38	8.67	38.60	-0.65	-2.11,	0.81
	F2iP	ng/ml	160	-0.28	0.56	0.92	4.98	-0.48	-2.09,	1.13
	F2iP-M	ng/ml	162	-0.13	0.77	0.39	2.05	-0.22	-1.66,	1.23

* N may vary due to missing values.

** Beta–coefficient from linear regression model adjusted for age and treatment for BPH

# Difference in PV or LUTS between the quartile values of each inflammatory marker. The interaction between IL-1β and BPH treatment on LUTS severity was marginally significant (p = 0.09). Difference in AUA-SI across inter-quartile range of IL-1b = -3.70 (-6.61, -0.80), among those receiving any BPH treatment (n = 45).

### Systemic Inflammation and Prostate Volume or LUTS severity

We analyzed a panel of four serum cytokines (IL-6, IL-8, TNF-α, and IL-1β) and three urine biomarkers (PGE-M, F2iP, F2iP-M) to investigate the relationship between systemic inflammation and oxidative stress markers with prostate volume or LUTS severity (Table 3). Prostate volume was not associated with levels of serum inflammatory cytokines. Urinary markers of oxidative stress tended to be associated with a smaller prostate volume, with a marginally significant inverse association between urinary F2iP levels and prostate volume (p = 0.08). We similarly found that urinary PGE-M was associated with a smaller prostate volume among men under BPH treatment (p-interaction = 0.015, Table C in S1 File).

LUTS severity was significantly lower with increasing blood IL-1β levels (p = 0.04), particularly among those men under BPH treatment such that adjusted AUA-SI scores were 3.70 lower at the upper vs. lower quartile of IL-1β within those men treated for BPH (Table D in S1 File: n = 45; -3.70, 95% CI (-6.61, -0.89), p<0.05). Other systemic inflammatory markers such as IL-6 and IL-8 tended to also have inverse relationships with LUTS severity but did not reach statistical significance.

### Prostate Tissue Inflammation and Prostate Volume or LUTS Severity

We next investigated the relationship between prostate tissue inflammation and BPH outcomes. Table 4 includes descriptive median values of prostate size or LUTS severity across levels of inflammation grade, location, and aggressiveness, as well as adjusted mean differences in prostate size or LUTS severity between inflammation scoring criteria. Adjusted mean prostate volume was somewhat greater with an increasing number of inflammatory regions and increasing grade of inflammation, but these differences were not statistically significant. Prostate volume also was not significant associated with inflammation grade, location, aggressiveness, or extent (Tables D and E in S1 File). In contrast, LUTS severity was significantly associated with the presence of severe inflammation grade, such that men with severe inflammation had a 7.07 higher adjusted AUA-SI score than men with mild inflammation (p = 0.004). LUTS severity scores were also a marginally significant 3.16 higher (p = 0.058) among men with inflammation regions in the non-glandular vs. glandular compartment.

**Table 4:** Properties of Prostate Tissue inflammation, and Association with Prostate Volume or LUTS Severity.

**Prostate Volume (mls)**							
			**Descriptive Values**		Adjusted Comparisons*		
**Prostate Tissue Inflammation**	**Level**	**n**	**Median**	**Quartiles**	**Difference**	**P**	**95% CI**
# of Inflammatory Regions	0	92	46.0	35.0 to 59.3	Reference		
	1	39	50.0	37.8 to 68.4	6.90	0.16	-2.73 to 16.5
	2 or more	55	51.0	30.8 to 77.5	4.09	0.35	-4.54 to 12.7
Grade	Mild	32	49.8	38.2 to 63.4	Reference		
	Moderate	50	53.3	31.0 to 78.6	3.52	0.58	-9.21 to 16.3
	Severe	12	45.0	28.9 to 87.9	5.66	0.55	-13.0 to 24.3
Location near Gland	Glandular	68	46.3	30.9 to 75.3	Reference		
	Non-glandular	26	56.7	49.0 to 76.8	4.14	0.52	-8.54 to 16.8
Aggressiveness	None/Minimal	51	50.0	37.8 to 78.0	Reference		
	< 25%	15	45.0	29.1 to 65.9	-7.52	0.31	-22.1 to 7.07
	> 25%	12	40.0	30.9 to 56.6	-7.98	0.32	-23.9 to 7.99
**LUTS (AUA-SI)**							
				**Descriptive Values**	**Adjusted Comparisons**		
**Prostate Tissue Inflammation**	**Level**	**n**	**Median**	**Quartiles**	**Difference**	**P**	**95% CI**
# of Inflammatory Regions	0	96	7.0	4.0 to 14.0	Reference		
	1	39	7.0	3.5 to 15.5	-0.37	0.78	-2.98 to 2.24
	2 or more	56	10.5	7.0 to 16.2	1.18	0.32	-1.14 to 3.51
Grade	Mild	32	7.5	5.0 to 11.3	Reference		
	Moderate	51	10.0	4.0 to 16.0	1.10	0.52	-2.17 to 4.19
	Severe	12	16.5	11.8 to 21.8	7.07	0.004	2.39 to 11.8
Location near Gland	Glandular	69	10.0	4.0 to 14.0	Reference		
	Non-glandular	26	11.0	7.0 to 18.0	3.16	0.058	-0.11 to 6.43
Aggressiveness	None/Minimal	52	10.5	3.8 to 17.0	Reference		
	< 25%	15	11.0	6.5 to 13.5	-0.53	0.80	-4.91 to 3.85
	> 25%	12	8.5	5.3 to 10.5	-1.59	0.51	-6.39 to 3.20

*Adjusted Comparisons were calculated as the difference (increase or decrease) in prostate volume or LUTS severity relative to the reference category, after controlling for BPH treatment and age in a linear model.

**Fig 2:**
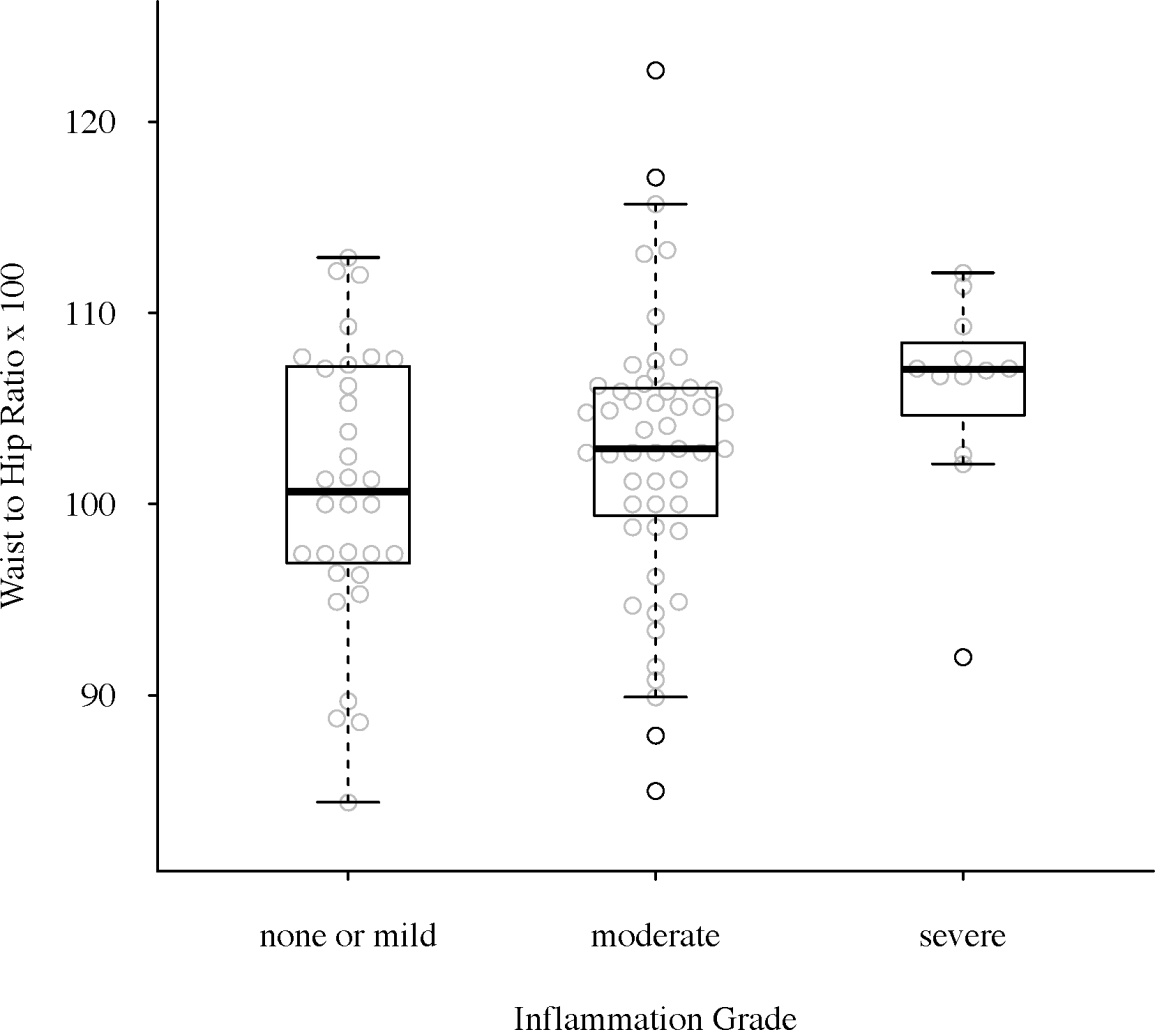
Prostate Tissue Inflammation and Waist-Hip Ratio: The grade of inflammation identified in prostate tissue was significantly associated with WHR, such that men with severe inflammation had a significantly higher WHR (p = 0.02; age and BPH treatment adjusted).

To consider the role of obesity on inflammation grade, we compared all obesity and body composition measures and against inflammation grade within the subset of participants with any prostate inflammation. Only WHR was significantly associated with inflammation grade, such that WHR increased with increasing inflammation grade (p = 0.02, adjusted for age and BPH treatment) (Fig 2). Analysis of the relationship between LUTS severity and WHR within this subset of participants also found that a higher WHR was significantly associated with moderate/severe LUTS (AUA-SI≥8: OR_(WHR>1.03)_ = 2.56, p = 0.03, adjusted for age and BPH treatment).

### Immune Cell Infiltration and Prostate Volume or LUTS severity

We scored the % of tissue as stained for CD3 or CD20 as indicators of T or B cell invasion, respectively (Table 5). To separate a broad effect across tissue from the possibility of a more aggressive local effect on LUTS and prostate volume, we separately scored average levels of staining across all tissue and the local region of tissue with the greatest immune cell staining within each participant. CD3 and CD20 positive staining were not significantly associated with prostate volume, although we note that increased maximal CD3 staining was marginally associated with a smaller prostate volume (p = 0.065). Furthermore, the presence of more intense CD20 positive staining was significantly associated with lower LUTS severity. No measure of obesity was significantly associated with either CD3 or CD20 scores in prostate tissue.

**Table 5:** Extent (% of tissue) of Inflammation, and CD3 and CD20 Immune Cell Infiltration, with Prostate Volume or LUTS severity.

**Prostate Volume (mls)**									
Inflammation	scale	N*	Beta**	P	Infl. Quartiles		PV Difference^#^	95% CI	
Extent (%) of inflammation	Average	94	-2.06	0.07	0.40	2.75	-4.84	-10.00,	0.31
	Maximum	94	-0.17	0.14	5.65	38.40	-5.52	-12.93,	1.89
CD3 positive Tissue	Average	107	-3.40	0.43	0.30	1.02	-2.46	-8.67,	3.74
	Maximum	107	-1.22	0.07	1.86	5.62	-4.60	-9.49,	0.30
CD20 positive Tissue	Average	107	16.01	0.33	0.007	0.13	1.96	-2.02,	5.94
	Maximum	107	0.70	0.60	0.073	1.91	1.29	-3.53,	6.10
**LUTS**									
Inflammation	scale	N	Beta	P	Infl. Quartiles		LUTS Difference	95% CI	
Extent (%) of inflammation	Average	95	-0.04	0.88	0.40	2.75	-0.10	-1.49,	1.28
	Maximum	95	-0.03	0.25	5.65	38.40	-1.15	-3.10,	0.81
CD3 positive Tissue	Average	108	1.11	0.30	0.30	1.02	0.81	-0.73,	2.34
	Maximum	108	0.14	0.41	1.86	5.62	0.52	-0.72,	1.76
CD20 positive Tissue	Average	108	-7.50	0.06	0.007	0.13	-0.92	-1.88,	0.05
	Maximum	108	-0.82	0.01	0.073	1.91	-1.50	-2.68,	-0.32

* N may vary due to missing values.

** Beta–coefficient from linear regression model adjusted for age and treatment for BPH

# Difference in PV or LUTS between the quartile values of each immune cell infiltration score, adjusted for age and treatment for BPH.

## Discussion

We investigated the role of inflammation in explaining the relationship between obesity and BPH outcomes, including LUTS severity and prostate enlargement. Overall, greater body mass, estimated by BMI, WHR, WC, %BF, fat mass, and blood leptin, was significantly associated with prostate enlargement but not with LUTS severity. Biomarkers of systemic inflammation were not consistently associated with a greater prostate size or more severe LUTS, and levels of prostate tissue inflammation were not associated with prostate enlargement. Severe inflammatory regions in the prostate, however, were significantly associated with LUTS severity. Furthermore, we found among men with prostate tissue inflammation that WHR was significantly associated with increasing grade of inflammation and also with more severe LUTS, consistent with a role of centralized fat deposition with LUTS severity.

### Obesity and BPH

Excess adipose tissue may affect inflammation, lipid metabolism, insulin resistance, and adipokine levels with a plausible role in prostate enlargement and LUTS pathophysiology. For example, IL-6 is produced by a number of cell types, including white adipose tissue where expression of IL-6 has a pro-inflammatory effect. We have previously shown that increased IL-6 levels are associated with BPH progression [33]. Such changes, resulting from a systemic insult, may increase the pro-inflammatory environment systemically and supporting pro-inflammatory signaling within prostate tissue. Two studies of male bariatric surgical patients found a reduction in LUTS severity after only one to three months of surgery [34, 35]. Similarly, a low-energy diet leading to a moderate weight loss was also associated with a reduction in LUTS severity [36], and further understanding the connection between weight and BPH outcomes may lead to alternative methods of symptom management.

Unfortunately, common research measures of obesity such as body mass index (BMI) are non-specific and do not provide insight to pathways or mechanisms involved [37]. Insight, however, may come from comparing different obesity measurements and BPH outcomes, and including biomarkers representing inflammatory pathways. We observed a broad and robust relationship between greater body size as estimated through multiple measurement approaches and prostate enlargement. Prostate enlargement is a significant cause for LUTS, but LUTS is not solely a function of prostate size but also involves weakness of the detrusor muscle in the bladder, infection, and dysregulation of the autonomic nervous system. In this analysis, the robust relationship between obesity and prostate enlargement did not extend to LUTS severity. Indeed, LUTS severity, unlike prostate enlargement, is as much, if not more, consistently associated with physical activity as with obesity, further illustrating a separation between prostate enlargement and LUTS and the outcome from multiple component causes [38–40].

### Systemic Inflammation

With increasing adiposity, there is an increase in lipid accumulation and adipocyte cell size, inducing hypoxia and cell necrosis. M1 macrophages invade the adipose tissue and release proinflammatory cytokines into circulation. Through measurement of listed blood and urinary biomarkers of inflammation and oxidative stress such as IL-6 and PGE-M, we investigated the role of systemic inflammation on prostate enlargement and LUTS severity [18, 19]. Schenk and colleagues reported that IL-6 levels were associated with LUTS severity in the PCPT, and controlling for BMI did not alter the association substantially [41]. In contrast, we did not see an association between IL-6 and prostate volume or increased LUTS severity. Indeed, cytokines such as IL-6 and IL-1β, as well as urinary PGE-M levels, tended to be associated with somewhat lower LUTS severity, with stronger effects among men under treatment for BPH. The exception may be leptin, since leptin is not only an adipocytokine but also enhances cytokine production and induces T cell activity [42]. However, the lack of association between prostate enlargement and other proinflammatory cytokines suggests that leptin, in this case, serves as an obesity biomarker rather than an inflammation biomarker. That pro-inflammatory cytokines were not related to prostate enlargement suggests a broader metabolic effect may be involved perhaps represented by the metabolic syndrome such as hyperlipidemia [38].

### Prostate Tissue Inflammation

Prostate tissue inflammation was associated with LUTS severity in MTOPS and REDUCE [8, 10]. However, we did not see an association between prostate size or LUTS severity with the number of regions of inflammation. Additionally, prostate size was not associated with inflammation grade, location, extent, or aggressiveness. In contrast, inflammation grade was significantly associated with LUTS severity, with those men with severe inflammatory regions reported an average 7 points higher on the AUA-SI. This highlights the importance of characterizing the properties of inflammatory regions. We further note that regions of inflammation distant from the glandular compartment had a nonsignificantly greater LUTS severity (p = 0.058), consistent with a remodeling of the stromal compartment of the prostate leading to hyperplasia and a potential target for future investigation.

WHR was significantly associated with increasing grade of inflammation, and with LUTS severity, suggesting that centralized adiposity as reflected by the WHR advances the severity of inflammatory regions leading to increased LUTS severity. Increasing centralized adipose deposition is a component of the broader metabolic syndrome, associated with inflammatory diseases including diabetes, hyperlipidemia, and cardiovascular disease. Indeed, the rapid reduction in LUTS severity following bariatric surgery and before maximal weight loss suggests some aspect of metabolic dysregulation such as insulin sensitivity could be involved [35]. Centralized adiposity also plays a stronger role than subcutaneous adiposity toward the activation of T cells, natural killer (NK) cells, and macrophages, and also affects lipid metabolism in the liver [43–45]. It is also possible that WHR reflects a measure of periprostatic adipose tissue and the local release of paracrine factors affecting prostate tissue. Whether NSAIDS [46], lipid medications [47], or weight reduction may reduce the burden of LUTS requires further investigation.

### Immune Cell Infiltration

Inflammatory infiltrates may include T and B lymphocytes [48, 49], however we did not see a strong or significant relationship between CD3 or CD20 immune cell infiltration with greater prostate volume or LUTS. If anything, greater CD3 or CD20 immune cell infiltration had an inverse association with prostate volume or LUTS, and indeed the identification of regions of more intense CD20 staining was significantly associated with lower LUTS severity. Inflammation is a common finding in prostate tissue, obtained either at biopsy or at the time of surgery, and our data demonstrate that the presence of inflammation alone is insufficient to affect LUTS severity. Indeed, the primary role of the immune system is to protect against a tissue challenge, and an alternative interpretation of our data would suggest that an inadequate CD20 response contributes toward the development of LUTS. Additionally, despite a presumed role of androgen activity in BPH, low androgen levels also have been correlated with greater prostate inflammation [50]. Such compensatory mechanisms may be why measuring androgen levels alone has been served to better understand BPH progression. We did not find that markers of immune cell infiltration were significantly associated with obesity indices, however, and thus we were not able to establish a link between obesity and an inadequate immune cell response in prostate tissue.

### Strengths and Limitations

Strengths in this analysis include the simultaneous evaluation of tissue, blood, and urine biomarkers of inflammation and oxidative stress. Rather than rely solely on BMI as our obesity measure, we also measured WC, WHR, blood leptin levels, and %BF and fat mass by bioelectric impedance analysis. All body size measures were obtained by trained staff, and prior to diagnosis to prevent any bias related to the knowledge of diagnosis on data collection, patient reporting, or treatment effects. We restricted our analysis to men without prostate cancer as determined by prostate biopsy to minimize the likelihood that LUTS derived from prostate cancer rather than BPH related conditions. Furthermore, inflammation scoring of prostate tissue was performed on biopsies from the left and right side of the prostate for each patient, and reviewed by a reference pathologist.

Study limitations include difficulty in determining temporal relationships between measurements within the cross-sectional study design. For example, it is possible that prostate size or LUTS altered in some way urinary biomarker levels, although it is unlikely that prostate size would affect BMI or body composition. BPH treatment could have affected biomarker values, however interaction terms were for the most part non-significant, analyses stratified by BPH treatment status did not suggest a consistent pattern or effect, and we control for BPH treatment throughout the main analysis. The blood biomarkers were assayed after several years storage at -80°C, which may have decreased precision. It was necessary to measure tissue inflammation in the peripheral zone of the prostate, however the transitional zone may have more direct impact on BPH outcomes. Difuccia and colleagues compared inflammatory cell density across prostate zones, and found peripheral zone inflammatory cell density correlated significantly with inflammatory cell density in transitional zone immune cell density [24]. We measured CD3 and CD20 to provide a score of T and B cell infiltration in prostate tissue, but understand that of other components of immune response may be involved. Tissue inflammation scoring by grade and extent would capture the total inflammatory effect across all immune cell types. We did not adjust for multiple testing, but instead used a traditional significance level of p<0.05 and interpreted results based on our *a priori* hypothesis. Also, there were several associations that had marginal statistical significance at p<0.10 and, given the imprecision of biomarker analysis, should be considered in future investigations The majority of study participants were white, and results may not generalize to other race/ethnicities.

### Conclusion

Centralized adipose deposition was associated with the severity of prostate tissue inflammation and LUTS within the subset of participants with prostate tissue inflammation. An approach to minimize centralized fat deposition may reduce LUTS severity in BPH patients. Lack of a clear relationship between blood or urinary biomarkers of inflammation or oxidative stress with prostate size or LUTS suggests the effects of obesity on BPH may be mediated by factors aside from systemic pro-inflammatory cytokines or oxidative stress. Further research is needed to expand the panel of pathways potentially affecting the obesity-BPH relationship to identify this mechanism.

## Supporting Information

S1 File Age-adjusted Interactions with BPH Treatment. (DOCX)

S2 File Minimal Dataset. (CSV)

## References

[1] Platz EA, Smit E, Curhan GC, Nyberg J, Giovannucci E. Prevalence of and racial/ethnic variation in lower urinary tract symptoms and noncancer prostate surgery in U.S. men. Urology. 2002;59(6):877–83. 12031373 10.1016/s0090-4295(01)01673-9

[2] Berry SJ, Coffey DS, Walsh PC, Ewing LL. The development of human benign prostatic hyperplasia with age. J Urol. 1984;132(3):474–9. 6206240 10.1016/s0022-5347(17)49698-4

[3] Rhodes T, Girman CJ, Jacobsen SJ, Roberts RO, Guess HA, Lieber MM. Longitudinal prostate growth rates during 5 years in randomly selected community men 40 to 79 years old. J Urol. 1999;161(4):1174–9. 10081864

[4] Ziada A, Rosenblum M, Crawford ED. Benign prostatic hyperplasia: an overview. Urology. 1999;53(3, Supplement 1):1–6. 10094094 10.1016/s0090-4295(98)00532-9

[5] McConnell JD, Roehrborn CG, Bautista OM, Andriole GL Jr., Dixon CM, Kusek JW, et al The Long-Term Effect of Doxazosin, Finasteride, and Combination Therapy on the Clinical Progression of Benign Prostatic Hyperplasia. The New England Journal of Medicine. 2003;349(25):2387–98. 14681504 10.1056/NEJMoa030656

[6] Isaacs JT, Coffey DS. Etiology and disease process of benign prostatic hyperplasia. The Prostate. 1989;2(Supplement):33–50. 2482772 10.1002/pros.2990150506

[7] Delongchamps NB, de la Roza G, Chandan V, Jones R, Sunheimer R, Threatte G, et al Evaluation of Prostatitis in Autopsied Prostates—Is Chronic Inflammation More Associated With Benign Prostatic Hyperplasia or Cancer? The Journal of Urology. 2008;179(5):1736–40. 10.1016/j.juro.2008.01.034 18343414 PMC2661538

[8] Roehrborn CG. Definition of at-risk patients: baseline variables. BJU Int. 2006;97 Suppl 2:7–11. 16507046 10.1111/j.1464-410X.2006.06098.x

[9] Kramer G, Marberger M. Could inflammation be a key component in the progression of benign prostatic hyperplasia? Curr Opin Urol. 2006;16(1):25–9. 16385197

[10] Nickel JC, Roehrborn CG, O'Leary MP, Bostwick DG, Somerville MC, Rittmaster RS. The Relationship between Prostate Inflammation and Lower Urinary Tract Symptoms: Examination of Baseline Data from the REDUCE Trial. Eur Urol. 2007. 10.1016/j.eururo.2007.11.026 PMC2643127 18036719

[11] Hammarsten J, Hogstedt B, Holthuis N, Mellstrom D. Components of the metabolic syndrome—risk factors for the development of benign prostatic hyperplasia. Prostate Cancer Prostatic Dis. 1998;1:157–62. 12496910 10.1038/sj.pcan.4500221

[12] Dahle SE, Chokkalingam AP, Gao YT, Deng J, Stanczyk FZ, Hsing AW. Body size and serum levels of insulin and leptin in relation to the risk of benign prostatic hyperplasia. J Urol. 2002;168(2):599–604. 12131317

[13] Kristal AR, Arnold KB, Schenk JM, Neuhouser ML, Weiss N, Goodman P, et al Race/ethnicity, obesity, health related behaviors and the risk of symptomatic benign prostatic hyperplasia: results from the prostate cancer prevention trial. J Urol. 2007;177(4):1395–400. 17382740 10.1016/j.juro.2006.11.065

[14] Giovannucci E, Rimm EB, Chute C, Kawachi I, Colditz GA, Stampfer M, et al Obesity and Benign Prostatic Hyperplasia. Am J Epidemiol. 1994;140(11):989–1002. 7527182 10.1093/oxfordjournals.aje.a117206

[15] Burke JP, Rhodes T, Jacobson DJ, McGree ME, Roberts RO, Girman CJ, et al Association of Anthropometric Measures with the Presence and Progression of Benign Prostatic Hyperplasia. Am J Epidemiol. 2006;164(1):41–6. 16611664 10.1093/aje/kwj151

[16] Parsons JK, Carter HB, Partin AW, Windham BG, Metter EJ, Ferrucci L, et al Metabolic Factors Associated with Benign Prostatic Hyperplasia. J Clin Endocrinol Metab. 2006;91(7):2562–8. 16608892 10.1210/jc.2005-2799 PMC2645661

[17] Fowke J, Munro H, Signorello L, Blot W, Penson D. Association Between Socioeconomic Status (SES) and Lower Urinary Tract Symptom (LUTS) Severity Among Black and White Men. J GEN INTERN MED. 2011;26(11):1305–10. 10.1007/s11606-011-1776-8 21720905 PMC3208454

[18] Cottam DR, Mattar SG, Barinas-Mitchell E, Eid G, Kuller L, Kelley DE, et al The chronic inflammatory hypothesis for the morbidity associated with morbid obesity: implications and effects of weight loss. Obes Surg. 2004;14(5):589–600. 15186624 10.1381/096089204323093345

[19] Yudkin JS. Adipose tissue, insulin action and vascular disease: inflammatory signals. Int J Obes. 2003;27:S25–S8. 10.1038/sj.ijo.0802496 14704740

[20] Begley LA, Kasina S, MacDonald J, Macoska JA. The inflammatory microenvironment of the aging prostate facilitates cellular proliferation and hypertrophy. Cytokine. 2008;43(2):194–9. 10.1016/j.cyto.2008.05.012 18572414 PMC2538565

[21] De Marzo AM, Platz EA, Sutcliffe S, Xu J, Gronberg H, Drake CG, et al Inflammation in prostate carcinogenesis. Nature Reviews Cancer. 2007;7(4):256–69. 17384581 10.1038/nrc2090 PMC3552388

[22] Fowke JH, Motley SS, Smith JA Jr., Cookson MS, Concepcion R, Chang SS, et al Association of nonsteroidal anti-inflammatory drugs, prostate specific antigen and prostate volume. J Urol. 2009;181(5):2064–70. 10.1016/j.juro.2009.01.031 19286210 PMC2679527

[23] Barry MJ, Fowler FJ Jr., O'Leary MP, Bruskewitz RC, Holtgrewe HL, Mebust WK, et al The American Urological Association symptom index for benign prostatic hyperplasia. The Measurement Committee of the American Urological Association. J Urol. 1992;148(5):1549–57. 1279218 10.1016/s0022-5347(17)36966-5

[24] Difuccia B, Keith I, Teunissen B, Moon T. Diagnosis of prostatic inflammation: Efficacy of needle biopsies versus tissue blocks. Urology. 2005;65(3):445–8. 15780352 10.1016/j.urology.2004.10.031

[25] Nickel JC, True LD, Krieger JN, Berger RE, Boag AH, Young ID. Consensus development of a histopathological classification system for chronic prostatic inflammation. BJU International. 2001;87(9):797–805. 11412216 10.1046/j.1464-410x.2001.02193.x

[26] Song L, Zhu Y, Han P, Chen N, Lin D, Lai J, et al A Retrospective Study: Correlation of Histologic Inflammation in Biopsy Specimens of Chinese Men Undergoing Surgery for Benign Prostatic Hyperplasia With Serum Prostate-specific Antigen. Urology. 2011;77(3):688–92. 10.1016/j.urology.2010.07.493 20974483

[27] Irani J, Goujon JM, Ragni E, Peyrat L, Hubert J, Saint F, et al High-grade inflammation in prostate cancer as a prognostic factor for biochemical recurrence after radical prostatectomy. Pathologist Multi Center Study Group. Urology. 1999;54(3):467–72. 10475356 10.1016/s0090-4295(99)00152-1

[28] Irani J, Levillain P, Goujon JM, Bon D, Dore B, Aubert J. Inflammation in benign prostatic hyperplasia: correlation with prostate specific antigen value. J Urol. 1997;157(4):1301–3. 9120926 10.1016/s0022-5347(01)64957-7

[29] Murphey LJ, Williams MK, Sanchez SC, Byrne LM, Csiki I, Oates JA, et al Quantification of the major urinary metabolite of PGE2 by a liquid chromatographic/mass spectrometric assay: determination of cyclooxygenase-specific PGE2 synthesis in healthy humans and those with lung cancer. AnalBiochem. 2004;334(2):266–75. 10.1016/j.ab.2004.08.019 15494133

[30] Morrow JD. The isoprostanes—unique products of arachidonate peroxidation: their role as mediators of oxidant stress. Curr PharmDes. 2006;12(8):895–902. 10.2174/138161206776055985 16533158

[31] Roberts LJ, Morrow JD. Measurement of F2-isoprostanes as an index of oxidative stress in vivo. Free Radical Biology and Medicine. 2000;28(4):505–13. 10719231 10.1016/s0891-5849(99)00264-6

[32] Nechuta S, Cai Q, Zheng Y, Milne G, Cai H, Dai Q, et al Urinary biomarkers of oxidative stress and breast cancer survival. Cancer Causes & Control. 2014;25(6):701–7. 24820618 10.1007/s10552-014-0373-7 PMC4031460

[33] Lin-Tsai O, Clark PE, Miller NL, Fowke JH, Hameed O, Hayward SW, et al Surgical intervention for symptomatic benign prostatic hyperplasia is correlated with expression of the AP-1 transcription factor network. Prostate. 2014;74(6):669–79. 10.1002/pros.22785 24500928 PMC4160824

[34] Groutz A, Gordon D, Schachter P, Amir H, Shimonov M. Effects of bariatric surgery on male lower urinary tract symptoms and sexual function. Neurourology and Urodynamics. 2016:n/a-n/a. 10.1002/nau.22980 26879634

[35] Luke S, Addison B, Broughton K, Masters J, Stubbs R, Kennedy-Smith A. Effects of bariatric surgery on untreated lower urinary tract symptoms: a prospective multicentre cohort study. BJU International. 2015;115(3):466–72. 10.1111/bju.12943 25265457

[36] Khoo J, Piantadosi C, Worthley S, Wittert GA. Effects of a low-energy diet on sexual function and lower urinary tract symptoms in obese men. Int J Obes. 2010;34(9):1396–403. 10.1038/ijo.2010.76 20404829

[37] Wang S, Mao Q, Lin Y, Wu J, Wang X, Zheng X, et al Body mass index and risk of BPH: a meta-analysis. Prostate Cancer Prostatic Dis. 2012;15(3):265–72. 10.1038/pcan.2011.65 22183774

[38] Russo GI, Castelli T, Urzi D, Privitera S, Fragala E, La Vignera S, et al Connections between lower urinary tract symptoms related to benign prostatic enlargement and metabolic syndrome with its components: a systematic review and meta-analysis. The aging male: the official journal of the International Society for the Study of the Aging Male. 2015;18(4):207–16. 10.3109/13685538.2015.1062980 26171768

[39] Fowke JH, Phillips S, Koyama T, Byerly S, Concepcion R, Motley SS, et al Association between physical activity, lower urinary tract symptoms (LUTS) and prostate volume. BJU Int. 2013;111(1):122–8. 10.1111/j.1464-410X.2012.11287.x 22726636 PMC3460041

[40] Fowke JH, Motley SS, Cookson MS, Concepcion R, Chang SS, Wills ML, et al The association between body size, prostate volume and prostate-specific antigen. Prostate Cancer Prostatic Dis. 2007;10(2):137–42. 17179979 10.1038/sj.pcan.4500924

[41] Schenk JM, Kristal AR, Neuhouser ML, Tangen CM, White E, Lin DW, et al Biomarkers of systemic inflammation and risk of incident, symptomatic benign prostatic hyperplasia: results from the prostate cancer prevention trial. American journal of epidemiology. 2010;171(5):571–82. 10.1093/aje/kwp406 20142396 PMC2842217

[42] Procaccini C, Jirillo E, Matarese G. Leptin as an immunomodulator. Molecular Aspects of Medicine. 2012;33(1):35–45. 10.1016/j.mam.2011.10.012 22040697

[43] Despres J-P, Lemieux I. Abdominal obesity and metabolic syndrome. Nature. 2006;444(7121):881–7. 17167477 10.1038/nature05488

[44] Hamdy O, Porramatikul S, Al-Ozairi E. Metabolic obesity: the paradox between visceral and subcutaneous fat. Current diabetes reviews. 2006;2(4):367–73. 18220642 10.2174/1573399810602040367

[45] O'Rourke RW. Inflammation in obesity-related diseases. Surgery. 2009;145(3):255–9. 10.1016/j.surg.2008.08.038 19231576 PMC2749322

[46] Schenk JM, Calip GS, Tangen CM, Goodman P, Parsons JK, Thompson IM, et al Indications For and Use of Nonsteroidal Antiinflammatory Drugs and the Risk of Incident, Symptomatic Benign Prostatic Hyperplasia: Results From the Prostate Cancer Prevention Trial. American journal of epidemiology. 2012;176(2):156–63. 10.1093/aje/kwr524 22759721 PMC3493197

[47] Penna G, Fibbi B, Amuchastegui S, Cossetti C, Aquilano F, Laverny G, et al Human Benign Prostatic Hyperplasia Stromal Cells As Inducers and Targets of Chronic Immuno-Mediated Inflammation. The Journal of Immunology. 2009;182(7):4056–64. 10.4049/jimmunol.0801875 19299703

[48] Steiner GE, Stix U, Handisurya A, Willheim M, Haitel A, Reithmayr F, et al Cytokine expression pattern in benign prostatic hyperplasia infiltrating T cells and impact of lymphocytic infiltration on cytokine mRNA profile in prostatic tissue. Laboratory investigation; a journal of technical methods and pathology. 2003;83(8):1131–46. 12920242 10.1097/01.lab.0000081388.40145.65

[49] Steiner GE, Newman ME, Paikl D, Stix U, Memaran-Dagda N, Lee C, et al Expression and function of pro-inflammatory interleukin IL-17 and IL-17 receptor in normal, benign hyperplastic, and malignant prostate. Prostate. 2003;56(3):171–82. 12772186 10.1002/pros.10238

[50] La Vignera S, Condorelli RA, Russo GI, Morgia G, Calogero AE. Endocrine control of benign prostatic hyperplasia. Andrology. 2016;4(3):404–11. 10.1111/andr.12186 27089546

