# Supplementary material for: Does Inflammation Mediate the Obesity and BPH Relationship? An Epidemiologic Analysis of Body Composition and Inflammatory Markers in Blood, Urine, and Prostate Tissue, and the Relationship with Prostate Enlargement and Lower Urinary Tract Symptoms
Source: PLoS One. 2016 Jun 23;11(6):e0156918. doi: 10.1371/journal.pone.0156918 (PMC4918934; doi:10.1371/journal.pone.0156918)
Supplement: S1 File — (DOCX) [file pone.0156918.s001.docx]

**Supplemental Tables**

**Table A: Interaction between Body Composition and BPH treatment on Prostate Volume; age adjusted**

|  | Unit | Quartiles | BPH TX | N | Difference in PV | 95 % CI | P-int |
| --- | --- | --- | --- | --- | --- | --- | --- |
| BMI | kg/m^2^ | 25.9 to 30.8 | Yes | 52 | 7.30 | -2.02 to 16.63 | 0.98 |
|  |  |  | No | 134 | 7.16 | 2.89 to 11.43 |  |
|  |  |  |  |  |  |  |  |
| WHR | 100 x (w/h) | 97.6 to 107.1 | Yes | 52 | 1.53 | -7.41 to 10.47 | 0.34 |
|  |  |  | No | 134 | 6.71 | 1.02 to 12.40 |  |
|  |  |  |  |  |  |  |  |
| WC | cm | 37.5 to 43.3 | Yes | 52 | 3.72 | -5.41 to 12.86 | 0.90 |
|  |  |  | No | 134 | 8.25 | 3.58 to 12.92 |  |
|  |  |  |  |  |  |  |  |
| Height | cm | 171.5 to 180.3 | Yes | 52 | -1.57 | -10.94 to 7.81 | 0.12 |
|  |  |  | No | 134 | 7.09 | 1.22 to 12.97 |  |
|  |  |  |  |  |  |  |  |
| % BF | % | 24.0 to 31.9 | Yes | 49 | 3.36 | -8.98 to 15.70 | 0.72 |
|  |  |  | No | 128 | 5.83 | 0.82 to 10.84 |  |
|  |  |  |  |  |  |  |  |
| Lean Mass | kg | 56.9 to 66.7 | Yes | 49 | 5.22 | -2.84 to 13.28 | 0.26 |
|  |  |  | No | 128 | 10.67 | 5.36 to 15.98 |  |
|  |  |  |  |  |  |  |  |
| Fat Mass | kg | 18.8 to 30.4 | Yes | 49 | 5.57 | -7.05 to 18.18 | 0.87 |
|  |  |  | No | 128 | 6.72 | 2.20 to 11.23 |  |

**Table B: Interaction between Body Composition and BPH treatment on Lower Urinary Tract Symptom Severity, age adjusted**

|  | Unit | Quartiles | BPH Tx | N | LUTS Difference | 95% CI | P-int |
| --- | --- | --- | --- | --- | --- | --- | --- |
| BMI | kg/m^2^ | 25.9 to 30.8 | Yes | 55 | -1.54 | -4.18 to 1.10 | 0.11 |
|  |  |  | No | 136 | 0.81 | -0.38 to 2.01 |  |
|  |  |  |  |  |  |  |  |
| WHR | 100 x (w/h) | 97.6 to 107.1 | Yes | 55 | -1.64 | -4.11 to 0.84 | 0.11 |
|  |  |  | No | 136 | 0.73 | -0.85 to 2.30 |  |
|  |  |  |  |  |  |  |  |
| WC | cm | 37.5 to 43.3 | Yes | 55 | -2.53 | -5.10 to 0.03 | 0.017 |
|  |  |  | No | 136 | 0.98 | -0.32 to 2.27 |  |
|  |  |  |  |  |  |  |  |
| Height | cm | 172 to 180 | Yes | 55 | -0.39 | -2.96 to 2.18 | 0.58 |
|  |  |  | No | 136 | 0.47 | -1.17 to 2.11 |  |
|  |  |  |  |  |  |  |  |
| % Body Fat | % | 24.0 to 31.9 | Yes | 52 | -0.45 | -3.73 to 2.83 | 0.58 |
|  |  |  | No | 130 | 0.55 | -0.84 to 1.94 |  |
|  |  |  |  |  |  |  |  |
| Lean Mass | kg | 56.9 to 66.7 | Yes | 52 | -1.03 | -3.33 to 1.28 | 0.10 |
|  |  |  | No | 130 | 1.25 | -0.30 to 2.80 |  |
|  |  |  |  |  |  |  |  |
| Fat Mass | kg | 18.8 to 30.4 | Yes | 52 | -1.19 | -4.71 to 2.33 | 0.31 |
|  |  |  | No | 130 | 0.76 | -0.51 to 2.02 |  |
|  |  |  |  |  |  |  |  |
| Leptin | Pg/ml | 4144 to 13417 | Yes | 45 | -1.13 | -4.18 to 1.93 | 0.27 |
|  |  |  | No | 117 | 0.80 | -0.67 to 2.26 |  |

**Table C: Interaction between biomarkers of Inflammation and BPH treatment on prostate volume, age adjusted**

|  |  | Unit | Quartiles | BPH TX | N | LUTS Difference | 95% CI | P-int |
| --- | --- | --- | --- | --- | --- | --- | --- | --- |
| Serum | IL-6 | Ng/ml | 1.90 to 6.17 | Yes | 48 | 2.19 | -9.73 to 14.10 | 0.82 |
|  |  |  |  | No | 115 | 0.71 | -4.77 to 6.18 |  |
|  |  |  |  |  |  |  |  |  |
|  | IL-8 | Ng/ml | 2.91 to 5.35 | Yes | 43 | -3.80 | -14.85 to 7.25 | 0.78 |
|  |  |  |  | No | 115 | -2.07 | -6.81 to 2.67 |  |
|  |  |  |  |  |  |  |  |  |
|  | TNF-α | Ng/ml | 2.83 to 5.03 | Yes | 43 | 1.63 | -7.79 to 11.05 | 0.94 |
|  |  |  |  | No | 115 | 2.03 | -3.27 to 7.32 |  |
|  |  |  |  |  |  |  |  |  |
|  | IL-1β | Ng/ml | 0.92 to 2.59 | Yes | 43 | -5.31 | -16.37to 5.75 | 0.53 |
|  |  |  |  | No | 115 | -1.25 | -7.34 to 4.84 |  |
|  |  |  |  |  |  |  |  |  |
|  | Leptin | pg/ml | 4144 to 13417 | Yes | 43 | 2.98 | -8.15 to 14.10 | 0.48 |
|  |  |  |  | No | 115 | 7.41 | 2.00 to 12.83 |  |
|  |  |  |  |  |  |  |  |  |
| Urine | PGE-M | Ng/ml | 8.67 to 38.60 | Yes | 45 | -15.22 | -26.48 to -3.96 | 0.015 |
|  |  |  |  | No | 115 | 0.61 | -5.46 to 6.67 |  |
|  |  |  |  |  |  |  |  |  |
|  | F2iP | Ng/ml | 0.92 to 4.98 | Yes | 45 | -4.96 | -15.14 to 5.22 | 0.90 |
|  |  |  |  | No | 111 | -4.17 | -11.45to 3.11 |  |
|  |  |  |  |  |  |  |  |  |
|  | F2iP-M | Ng/ml | 0.39 to 2.05 | Yes | 45 | -7.47 | -17.45 to 2.52 | 0.33 |
|  |  |  |  | No | 113 | -1.62 | -8.17 to 4.92 |  |

**Table D: Interaction between biomarkers of inflammation with BPH treatment on LUTS severity, age adjusted**

|  |  | Unit | Quartiles | BPH TX | N | Difference in PV | 95% CI | P-int |
| --- | --- | --- | --- | --- | --- | --- | --- | --- |
| Serum | IL-6 | Ng/ml | 1.92 to 6.17 | Yes | 45 | -2.67 | -5.77 to 0.42 | 0.32 |
|  |  |  |  | No | 117 | -0.94 | -2.40 to 0.53 |  |
|  |  |  |  |  |  |  |  |  |
|  | IL-8 | Ng/ml | 2.91 to 5.35 | Yes | 45 | -2.27 | -5.20 to 0.66 | 0.38 |
|  |  |  |  | No | 117 | -0.85 | -2.13 to 0.42 |  |
|  |  |  |  |  |  |  |  |  |
|  | TNF-α | Ng/ml | 2.83 to 5.03 | Yes | 45 | 0.12 | -2.43 to 2.67 | 0.99 |
|  |  |  |  | No | 117 | 0.14 | -1.19 to 1.46 |  |
|  |  |  |  |  |  |  |  |  |
|  | IL-1β | Ng/ml | 0.92 to 2.59 | Yes | 45 | -3.70 | -6.61 to -0.89 | 0.09 |
|  |  |  |  | No | 117 | -0.81 | -2.41 to 0.81 |  |
|  |  |  |  |  |  |  |  |  |
|  | Leptin | pg/ml | 4144 to 13417 | Yes | 45 | -1.13 | -4.18 to 1.93 | 0.27 |
|  |  |  |  | No | 117 | 0.80 | 0.67to 2.26 |  |
|  |  |  |  |  |  |  |  |  |
| Urine | PGE-M | Ng/ml | 8.67 to 38.60 | Yes | 47 | 1.52 | -1.57 to 4.62 | 0.12 |
|  |  |  |  | No | 117 | 1.25 | -2.92 to 0.42 |  |
|  |  |  |  |  |  |  |  |  |
|  | F2iP | Ng/ml | 0.92 to 4.98 | Yes | 47 | 1.42 | -1.37 to 4.21 | 0.11 |
|  |  |  |  | No | 113 | -1.36 | -3.35 to 0.63 |  |
|  |  |  |  |  |  |  |  |  |
|  | F2iP-M | Ng/ml | 0.39 to 2.05 | Yes | 47 | 1.64 | -1.02 to -1.02 | 0.11 |
|  |  |  |  | No | 115 | -0.95 | -2.69 to 0.79 |  |

**Table E: Interaction between Extent of inflammation, or CD3/CD20 infiltration, with BPH treatment on Prostate volume, age adjusted**

|  |  | Quartiles | BPH TX | N | PV Difference | 95% CI | P-int |
| --- | --- | --- | --- | --- | --- | --- | --- |
| Extent of inflammation | Average | 0.40 to 2.75 | Yes | 30 | -6.83 | -16.5 to 2.88 | 0.63 |
|  |  |  | No | 64 | -4.09 | -10.1 to 1.96 |  |
|  |  |  |  |  |  |  |  |
| Extent of inflammation | Maximum | 5.65 to 38.4 | Yes | 31 | -7.91 | -21.3 to 5.47 | 0.67 |
|  |  |  | No | 64 | -4.46 | -13.4 to 4.47 |  |
|  |  |  |  |  |  |  |  |
| CD3 positive Tissue | Average | 0.30 to 1.02 | Yes | 32 | 4.71 | -7.21 to 16.63 | 0.17 |
|  |  |  | No | 75 | -5.04 | -12.28 to 2.13 |  |
|  |  |  |  |  |  |  |  |
| CD3 positive Tissue | Maximum | 1.86 to 5.62 | Yes | 32 | -4.04 | -16.58 to 8.51 | 0.92 |
|  |  |  | No | 75 | -4.70 | -10.04 to 0.64 |  |
|  |  |  |  |  |  |  |  |
| CD20 positive Tissue | Average | 0.007 to 0.13 | Yes | 32 | 3.33 | -2.76 to 9.43 | 0.56 |
|  |  |  | No | 75 | 0.93 | -4.35 to 6.20 |  |
|  |  |  |  |  |  |  |  |
| CD20 positive Tissue | Maximum | 0.073 to 1.91 | Yes | 32 | 4.47 | -3.53 to 12.48 | 0.33 |
|  |  |  | No | 75 | -0.56 | -6.64 to 5.52 |  |

**Table E: Interaction with BPH treatment on LUTS severity, age adjusted**

|  |  | Quartiles | BPH | N | LUTS Difference | 95% CI | P-int |
| --- | --- | --- | --- | --- | --- | --- | --- |
|  |  |  |  |  |  |  |  |
| Extent of inflammation | Average | 0.40 to 2.75 | Yes | 31 | -0.73 | -3.36 to 1.90 | 0.59 |
|  |  |  | No | 64 | 0.11 | -1.53 to 1.75 |  |
|  |  |  |  |  |  |  |  |
| Extent of inflammation | Max | 5.65 to 38.4 | Yes | 31 | -2.52 | -6.08 to 1.03 | 0.36 |
|  |  |  | No | 64 | -0.56 | -2.94 to 1.82 |  |
|  |  |  |  |  |  |  |  |
| CD3 positive Tissue | Average | 0.30 to 1.02 | Yes | 33 | 1.26 | -1.71 to 4.24 | 0.48 |
|  |  |  | No | 77 | 0.029 | -1.80 to 1.86 |  |
|  |  |  |  |  |  |  |  |
| CD3 positive Tissue | Maximum | 1.86 to 5.62 | Yes | 33 | 2.45 | -0.69 to 5.59 | 0.25 |
|  |  |  | No | 77 | 0.44 | -0.91 to 1.79 |  |
|  |  |  |  |  |  |  |  |
| CD20 positive Tissue | Average | 0.007 to 0.13 | Yes | 33 | -0.56 | -2.10 to 0.99 | 0.39 |
|  |  |  | No | 77 | 0.33 | -0.99 to 1.64 |  |
|  |  |  |  |  |  |  |  |
| CD20 positive Tissue | Maximum | 0.073 to 1.91 | Yes | 33 | -0.27 | -2.29 to 1.75 | 0.31 |
|  |  |  | No | 77 | 1.03 | -0.49 to 2.56 |  |
